# Microfluidic Techniques for Analytes Concentration

**DOI:** 10.3390/mi8010028

**Published:** 2017-01-22

**Authors:** Cunlu Zhao, Zhengwei Ge, Chun Yang

**Affiliations:** 1 Key Laboratory of Thermo-Fluid Science and Engineering of MOE, School of Energy and Power Engineering, Xi’an Jiaotong University, Xi’an 710049, China; 2 School of Mechanical and Aerospace Engineering, Nanyang Technological University, 50 Nanyang Avenue, Singapore 639798, Singapore; GEZH0001@e.ntu.edu.sg

**Keywords:** microfluidics, sample preconcentration, stacking, field gradient focusing, electrokinetic trapping, immunocapture based trapping

## Abstract

Microfluidics has been undergoing fast development in the past two decades due to its promising applications in biotechnology, medicine, and chemistry. Towards these applications, enhancing concentration sensitivity and detection resolution are indispensable to meet the detection limits because of the dilute sample concentrations, ultra-small sample volumes and short detection lengths in microfluidic devices. A variety of microfluidic techniques for concentrating analytes have been developed. This article presents an overview of analyte concentration techniques in microfluidics. We focus on discussing the physical mechanism of each concentration technique with its representative advancements and applications. Finally, the article is concluded by highlighting and discussing advantages and disadvantages of the reviewed techniques.

## 1. Introduction

Microfluidics is a set of technologies that are capable of accurately manipulating a small amount of fluids and analytes with their volume ranging from microliters to picoliters. Particularly, microfluidics is the core technique for micro total analysis systems (µTAS) or so-called “lab on a chip” (LOC) systems which, during the past two decades, have had a revolutionary impact on biotechnology, medicine, optics and chemistry etc. [1,2,3]. These microfluidics-based analytical systems show great advantages compared to traditional analytical systems/devices: they consume extremely small amount of sample material and reagents; they can be inexpensive and disposable; the analysis time with microfluidic analytical systems tends to be very short; more prominently one single microfluidic analytical system usually can perform multiple sample manipulation functions, such as sample concentration, separation, detection, pumping, mixing, and chemical reactions etc.

One of the major challenges faced by microfluidics is the difficulty in detecting very dilute concentration of analytes (e.g., pathogen in saliva) with ultrasmall volumes of samples in microchannels. The poor detection sensitivity results from the extremely small quantities of dilute analytes and the very short (10–100 µm) optical detection path lengths [4]. In chemical or biochemical analyses such as the detection of drug molecules in biological fluids, the drug and its metabolites are usually at much lower concentration than those prepared in laboratory [5]. The miniaturized medical/biomedical analysis systems, serving as promising tools for fast clinical and forensic diagnostics, also demand the development of detection and sensing techniques to meet the requirements of sensitivity, resolution and reliability [6]. In the field of portable water analysis and environmental analysis of nonsaline and saline waters, the analysis equipment is not sensitive enough to detect the low concentrations of organic and inorganic substances present in the samples. For instance, the concentration of organomercury or organolead compounds is less than 1 ng/L in seawater, and the concentration of polyaromatic hydrocarbons is less than 1 nM in waste water [7].

There have been well-developed techniques for analyte concentration for large-volume liquid samples in environment analyses. For example, the extraction method is the most widely used in concentrating environmental samples. The idea of this technique is that it uses a material phase or extraction agent that has strong affinity (either physical or chemical) to a specific component in the sample to increase the concentration of that specific component. After many years’ development, it has numerous variations, such as liquid-liquid extraction (LLE), solid phase extraction (SPE), solid phase microextraction (SPME), magnetic solid phase extraction (MSPE), microwave-assisted extraction (MAE), supercritical fluid extraction (SFE) etc. These techniques have been successfully used in concentrating inorganic (largely heavy metal ions) and organic pollutants in large volumes of water sample. The state-of-the-art of the extraction methods has been well documented in Refs. [8,9,10,11]. Yet, concentration of analyte in ultra-small volume of sample (usually nanoliter), which is typically required in microfluidic applications, is still in the initial stage of development and facing various challenges. For example, capillary electrophoresis (CE) is a widely-used microfluidic separation technique in chemical, biological and medical analyses. During its development in more than 20 years, enhancing its selectivity and sensitivity for detection/separation is constantly an important topic. CE has two types of detection limit: the concentration detection limit and the mass detection limit. The concentration detection limit is defined as the minimum concentration which can be detected by a given detection technique: typical values for ultraviolet (UV) detection, electrochemical/fluorescence detection and optimized laser-induced fluorescence (LIF) detection are 10^−6^ M, 10^−8^ M and 10^−10^ M, respectively. The mass detection limit is the absolute mass subjected to the analytical separation. In capillary electrophoresis, absolute mass is in a quite small range from pico- to atto-mole. This is because in CE system, the total volumes handled are less than 10 microliters and injection volumes are in the range of several nanoliters or even picoliters [12]. Although some methods of detection, such as LIF and mass spectroscopy can potentially offer improved detection sensitivity, they are not widely applicable and also rather expensive. A more practical method of improving the sensitivity of CE is to increase the concentration of analytes for the analysis. Sample preconcentration methods, usually on-capillary, are used to concentrate dilute samples for increasing the CE sensitivity [13]. 

Hence, miniaturization of analytical devices involving micro/nanoliter samples call for the demand for efficient preconcentration or focusing of dilute samples prior to detection or further analyses. In microfluidics, numerous techniques have been developed to concentrate analytes for subsequent detection and manipulations. In this review, we provide an overview of microfluidic techniques for analyte concentration. According to the difference in physical mechanisms, this review categorize the concentration techniques into three major groups: the first group of concentration techniques termed as the stacking methods includes the sweeping method, the field amplified sample stacking (FASS) method and the isotachophoresis (ITP); the second group of analyte concentration methods is termed as the equilibrium field gradient focusing methods, including the isoelectric focusing (IEF) by creating a pH gradient, electric field gradient focusing (EFGF) by imposing an electric field gradient, and temperature gradient focusing (TGF) by using a temperature gradient; the third group includes some other concentration techniques based on various trapping mechanisms, including electrokinetic trapping (EKT), dielectrophoretic trapping (DEPT), immunocapture based trapping (ICBT), magnetic beads assisted trapping (MBAT), thermophoretic trapping (TPT) and some other emerging methods. The above three groups are reviewed in a sequential order; for each group the review begins with the discussion of its underlying concentration mechanism and application background, and subsequently presents some of its typical application examples. Finally, the review is concluded by summarizing advantages and disadvantages of the reviewed techniques.

## 2. Stacking Methods

**Figure 1:**
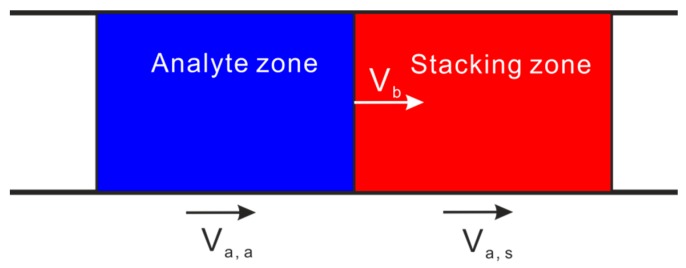
Schematics of the stacking mechanism via manipulation of the net velocities of analytes. The analyte in the analyte (stacking) zone moves with a velocity of *V*_a,a_ (*V*_a,s_), and the boundary between analyte zone and stacking zone moves with a velocity of *V*_b_.

The main principle of stacking methods is based on the mass conservation in electrophoresis with manipulation of net migration velocities of analytes. Figure 1 shows a schematic view of the stacking mechanism. The stacking zone and the analyte zone are separated by their common boundary. The analyte net velocities in the analyte zone and the stacking zone are denoted by *V*_a,a_ and *V*_a,s_, respectively. The velocity of the common boundary is *V*_b_. By manipulation of the magnitude of these three velocities, three cases can be distinguished [14], including boundary stacking when (*V*_a,s_ − *V*_b_)/( *V*_a,a_ − *V*_b_) < 0, proportional stacking when 0 < (*V*_a,s_ − *V*_b_)/( *V*_a,a_ − *V*_b_) < 1, and no stacking when (*V*_a,s_ − *V*_b_)/( *V*_a,a_ − *V*_b_) > 1. Clearly, proportional stacking and boundary stacking are two basic modes of stacking. In the proportional stacking, the analytes migrate into a stacking zone with decreasing migration velocity. In the boundary stacking, the analyte is focused at a sharp moving boundary, since the analytes in front of the boundary move slower than the sharp boundary and the analytes behind the boundary move faster than the sharp boundary. 

### 2.1. Sweeping

**Figure 2:**
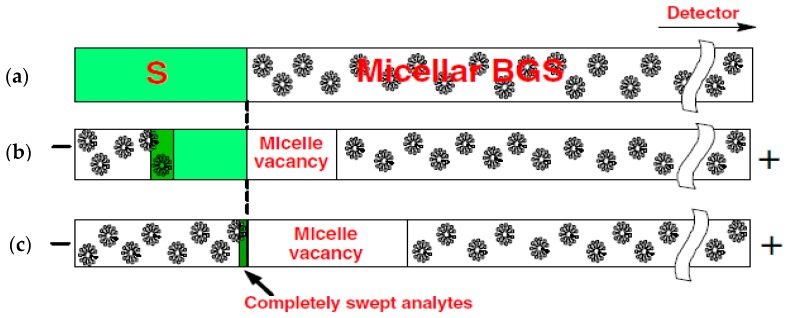
Principle of the sweeping in a homogenous electric field. (**a**) At beginning, the capillary is filled with analyte solution (S) and micellar background solution (BGS), and the two portions have a similar conductivity. (**b**) Application of a voltage electrophoretically drives the pseudo-stationary phase (PSP) into the S zone, and at the same time the moving PSP sweeps analyte molecules to compress the S zone. (**c**) As PSP completely fills the S zone, the analytes are completed swept to form a concentrated band. Reprint from Ref. [17].

Sweeping was initially developed for concentrating neutral hydrophobic analytes through picking and accumulation of analyte molecules in their pseudo-stationary phase (PSP) that penetrates the sample zone. It was first described in electrokinetic chromatography using a charged pseudo-stationary phase (i.e., micelle) [15]. In combination with stacking by ion selective injection, sweeping has also been applied to charged hydrophobic analytes [16,17]. To elucidate the sweep mechanism, an example of neutral analytes with a negatively charged PSP is shown in Figure 2. Micelles are chosen as the PSP and are suspended in the background solution (BGS). The conductivities of liquids are similar, and thus a homogenous electric field is expected in the capillary. Electroosmotic flow is neglected here, and neutral analytes have to be incorporated into charged micelles for transportation. Under such circumstances, neutral analytes are transported to the detector by micelle electrophoresis.

In Figure 2a, the analytes (S, the light shade area) containing no micelles are injected into a capillary filled with the BGS containing micelles. The analyte zone has the same conductivity as the BGS zone, which ensures a homogenous electric field along the capillary. In Figure 2b, as soon as a voltage is applied across the capillary (with both ends immersed in micellar BGS), the negatively charged micelles enter the capillary from the cathodic end due to electrophoresis. The moving micelles pick up the analytes in the S zone, and then the analytes accumulate into a narrow zone. The accumulation zone denoted by the dark shade area has a higher analyte concentration than the original analyte zone (light shade area). At the interface (dashed line) between the S and BGS zones, a micelle vacancy zone develops simultaneously. In Figure 2c, the micelles completely sweep all analytes into a much narrower zone. The analyte concentration after sweeping is predicted to be enhanced with a factor 1 + *k*, where *k* is the retention factor. Sweeping is capable of achieving a very high degree of sample concentration up to 5000-fold. The technique is useful only for small hydrophobic analytes with a high affinity to a mobile micellar phase [16,18].

The applications of sweeping to the preconcentration of various analytes have been achieved, such as phenols and pesticides in food samples and water, illicit drugs in urine samples, active ingredients of pharmaceutical samples, and even alkaloids in Chinese herbal medicines. The reviews [17,19] provide a good summary of diverse analytes that have been concentrated with the sweeping method. The sweeping method is also combined with other concentration techniques, such as dynamic pH junction and field amplified sample injection, to produce an improved concentration enhancement [17,18]. The combination of sweeping and field amplified sample injection produces a 10,000,000-fold increase in detection sensitivity for capillary electrophoresis separation, which is an unprecedentedly high value [20].

### 2.2. Field Amplified Sample Stacking (FASS)

Field-amplified sample stacking (FASS) is a commonly used method for preconcentrating analytes prior to capillary electrophoresis separation [21]. In FASS, the sample is prepared in the solution with a lower conductivity than the running buffer solution. One portion of capillary is filled with the sample solution of low-conductivity, while the remaining portion of capillary is filled with the running buffer of high-conductivity. Thus, the contact of high and low conductivity solutions inside a capillary creates a boundary. The electric field strength, as well as the electrophoretic velocities of the analytes in the low-conductivity zone is higher than that in the high-conductivity zone due to the difference in solution conductivities between the two zones. The velocity of analytes decreases when the analytes are transported from the low-conductivity zone to the high-conductivity zone. Hence, the analytes accumulate into a narrow concentrated band in the high-conductivity zone. The sample is concentrated due to the velocity change when it passes through the boundary between low- and high-conductivity zones. FASS can achieve fairly high concentration factors of about 100 to 1000 folds, but it requires multiple buffers [22]. 

Zhang and Thormann [23] demonstrated a head-column FASS for analysis of positively charged, hydrophobic compounds in capillary electrophoresis. To ensure the success of this technique: a water plug with length larger than 1 mm was introduced at the capillary inlet prior to injection of solutes; a modestly high voltage (<20 kV) was applied for less than 60 s during sample introduction; sample solutions with low-conductivity were employed. As a demonstration of the effectiveness of technique, the sensitivity in analyzing amiodarone and desethylamiodarone was enhanced over 1000-fold, as compared to that achieved by hydrodynamic injection without sample stacking. However, head-column FASS suffers from some disadvantages: biological samples have to contain no salts; the devices should be carefully designed to enable the easy application of voltage and pressure/vacuum during any cycle of the run.

How to setup a high conductivity gradient boundary is challenging in a practical FASS. To resolve this issue, Jung et al. [24] developed a novel FASS-capillary electrophoresis chip which uses a photo-initiated porous polymer structure to suppress the flow, but allows electromigration of sample analytes. This can make the sample injection and flow control for high-gradient FASS much easier. The technique yielded an 1100-fold increase of signal intensity in separation of fluorescein and Bodipy.

In order to investigate the dynamics at an interface between high- and low-conductivity zones in FASS, Bharadwaj and Santiago [25] theoretically modeled the FASS process as a combination of electromigration, diffusion, and advection of the multi-sample species and background electrolyte ions. Electric field, background electrolyte concentration, and sample-ion distribution were numerically simulated to predict the time evolution of the FASS process. The model was validated by on-chip FASS experiments. Sustarich et al. [26] studied the FASS in nanofluidic channels with electric double layer effects. Results showed that the concentration enhancement is better in nanochannels than in microchannels, but the inherent signals are relatively low due to the smaller amount of analytes handled in nanoscale level. 

**Figure 3:**
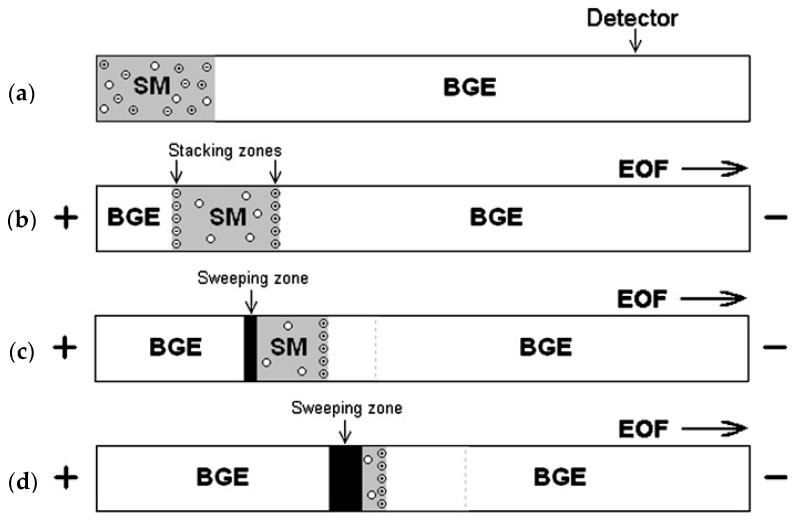
A FASS–sweeping combined mechanism for sample concentration. (**a**) Sample is loaded into the low conductivity sample matrix (SM) which has a lower pH value than the background electrolyte (BGE) that contains anionic micelles. (**b**) Under the effect of an applied voltage, charged analytes are stacked to the two boundaries between SM and BGE, and BGE with micelles enters the anodic end of the capillary by electroosmotic flow (EOF). (**c**) Movement of the micelles into the SM sweep sample compounds. (**d**) All sample compounds are completely swept. ⊖—Anionic analyte, ⊕—cationic analyte, ◯—neutral analyte. Reprint from Ref. [28].

For simultaneous concentration of multiple analytes, FASS needs to be combined with other concentration methods. One example is the FASS-sweeping combined technique [27,28]. Figure 3 shows the concentration enhancement of three analytes, namely positively charged, negatively charged and neutral, by a FASS-sweeping combined mechanism. Initially, the sample is loaded into the capillary (Figure 3a). The left portion of capillary is occupied by sample matrix (SM) which holds the sample and the rest is filled with back ground electrolyte (BGE) which contains anionic micelles. The application of a high voltage causes the stacking of charged analytes on the zone boundaries between BGE and SM (Figure 3b). At the same time, background electrolyte mixed with micelles, enters the capillary because of electroosmotic flow (EOF), and sweeps the sample zone analytes (Figure 3c). Thus, charged analytes are subject to both stacking and sweeping, while neutral analytes are only subject to sweeping. Finally, all analytes are completely swept (Figure 3d).

FASS is the simplest sample concentration technique used in capillary electrophoresis, and therefore has been employed in diverse fields, such as biology, environmental monitoring, food and pharmaceutical analyses. The readers can have more details from the recent review [19].

### 2.3. Isotachophoresis (ITP)

Isotachophoresis (ITP) is a technique using variable ion mobility zones for sample focusing and separation [29]. Three electrolytes are used in ITP: a leading electrolyte (LE), a trailing electrolyte (TE) and a sample analytes sandwiched between the two. As to the composition of LE and TE, ITP requires that LE and TE usually have a common counterion, but different coions. Coions (counterions) have an electric polarity same as (opposite to) the charged analytes. The ionic mobility of analytes should be higher than that of coions of the TE, but lower than that of coions of the LE. As an electric potential is applied across the entire zone, a low (high) electrical field in LE (TE) is generated. Analyte ions in the TE migrate faster than the coions of TE, while analytes in the LE migrate slower. Such analyte velocity difference in LE and TE results in the focusing of analytes at the LE/TE interface.

Being a sensitive analytical technique, the analytical capillary ITP has its advantages in concentrating and separating samples with large ratios of macrocomponents to microcomponents. Such samples can be analyzed with pretreatment by applying ITP alone or even better in combination with capillary zone electrophoresis or another separation method [30]. Ma et al. [31] performed an ITP preconcentration followed by capillary zone electrophoresis separation on a quartz microchip with UV detection. Compared to the sole capillary zone electrophoresis mode, the analysis sensitivity is increased 32-fold. Jung et al. [32] conducted a systematic experimental investigation of the initial sample ion concentration, leading ion concentration, and trailing ion concentration effects on ITP stacking. The single-column ITP was greatly improved with high leading ion concentration and electroosmotic flow suppression to achieve a million-fold concentration enhancement. Additionally, the single-column ITP can be readily integrated with capillary electrophoresis separation. Jung et al. [33] subsequently presented another highly sensitive capillary electrophoresis setup with combination of transient, single-interface on-chip ITP and a laser-induced confocal fluorescence detection. Effects of microscope objective specifications and intensity of excitation laser were studied experimentally to optimize the performance of setup. The new setup was demonstrated to separate and detect 100 aM fluorophores from the samples, which is the highest sensitivity ever reported for electrophoretic separation at that time.

Khurana and Santiago [34] developed a theoretical model to analyze the concentration enhancement with ITP peak mode. The model was optimized to achieve the maximum values of preconcentration ratio and peak analyte concentration by perfect suppression of electroosmotic flow. Bottenus et al. [35,36] concentrated the cardiac biomarker, cardiac troponin I (cTnI), using cationic ITP in a 3.9 cm long PMMA microfluidic channel. An increase in concentration by over a factor of more than 10,000 was observed due to the combination of ITP stacking and the reduction in cross-sectional area. Then, a 3-D numerical simulation of ITP was performed by the same group to predict the concentration enhancement of proteins in an ITP experiment, and this could be the first example of an ITP simulation in three dimensions [37]. Although ITP is a very powerful technique to concentrate and separate solutes, one apparent drawback is that it requires some priori knowledge of electrophoretic mobilities of sample ions.

## 3. Gradient Focusing Methods

**Figure 4:**
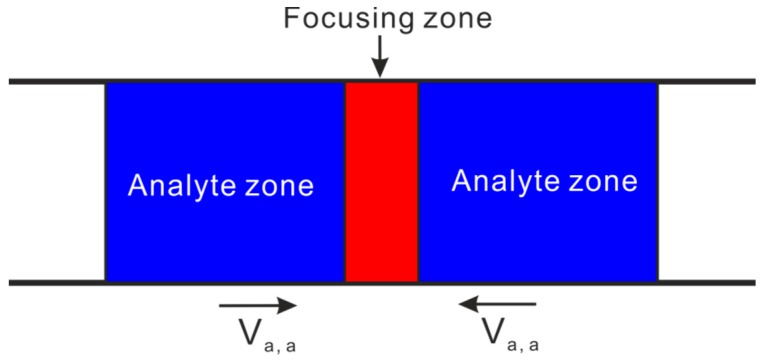
Schematic illustration showing of the gradient focusing mechanism. The analyte accumulates with a velocity of *V*_a,a_ towards the stationary focusing zone from two analyte zones.

The central idea of gradient focusing methods is explained in Figure 4. The analyte inside the two analyte zones moves with a velocity of *V*_a,a_ towards the focusing zone which is stationary, and gradually the analyte concentration inside the focusing zone is enhanced. To create a variation of analyte velocity in Figure 4 is critical for the success of analyte focusing, and usually various field gradients (such as pH gradient, electric field gradient and temperature gradient) are utilized for such purpose. Gradient focusing is different from stacking, although they both achieve an increase in analyte concentration by varying analyte axial velocity. The main difference between the two is the ways in which analytes are transported to the concentration region [38]. In the stacking, analytes are concentrated at a boundary because of reducing the magnitude of the analyte velocity across the boundary but no change of analyte velocity direction. Therefore, the extent of concentration enhancement is limited by such velocity reduction. However, for the focusing techniques, analytes are concentrated at a focusing point since analytes migrate to this point from both directions due to a change in direction of the analyte velocity. As a result, the concentration process continues at the focusing point provided that the focusing field is present or until the sample analyte is completely exhausted. The concentration enhancement in focusing techniques is determined by the velocity at which analytes migrate to the focusing zone and the duration for applying the focusing field.

### 3.1. Isoelectric Focusing (IEF)

Isoelectric focusing (IEF), also known as electrofocusing, is commonly used for concentrating and separating proteins at their respective isoelectric points in the presence of a pH gradient [39,40]. When protein molecules are subject to a pH gradient, they acquire different electric charge. At a specific value of pH (so called isoelectric point), the proteins are unchanged, while the proteins below and above such specific pH value are oppositely charged. Under the effect of an electric field, proteins move electrophoretically towards the isoelectric point and eventually stop at this point. Gradually, protein molecules become more concentrated at the isoelectric point. Successful implementation of IEF relies on the generation of stable pH gradients [41]. However, from an application viewpoint, IEF is limited due to the restriction of sample analytes with an accessible isoelectric point along a pH gradient [42]. In addition, the low solubility of most proteins at their isoelectric points highly degrade concentration enhancement by IEF [43].

Dynamic isoelectric focusing (DIEF) [44] is an improved capillary isoelectric focusing technique which is capable of controlling the shape of the electric field inside the capillary by additional high-voltage power supplies. DIEF is similar to capillary isoelectric focusing (cIEF), because proteins in both methods are focused at their isoelectric point by generating a pH gradient. The difference between cIEF and DIEF is that in cIEF the pH gradient and the electric field are constant, giving each focused protein in the sample a fixed position, while in DIEF, the pH gradient can be adjusted by manipulation of the electric field, making the control of position and width of each focused protein band available. By changing electric fields in DIEF, the protein band can be relocated to a desired location for collection and further analysis without the loss of focusing. The high peak capacity as well as the flexibility to control the focusing band width and location demonstrates that DIEF can be potentially a first dimension in a multidimensional separation system. The highest peak capacity in DIEF can be over 1000, as is evidenced by both mass spectrometry and direct imaging. 

Mosher and Thormann [45] performed a high-resolution computer simulation of the dynamics of IEF to search for more realistic input parameters for carrier ampholytes. The simulated cationic migrations were compared well with the experimental data. It was found that the cathodic migration makes the acidic portion of the gradient flat and the basic part steep, which is an additional argument disproving the notion that the focused zones of carrier ampholytes have no electrophoretic flux. Yang et al. [46] developed a free-flow IEF microfluidic device with glass coating by sol-gel methods to improve the bonding strength, chemical resistance and durability for reuse, as well as to supply a stable pH gradient.

### 3.2. Electric Field Gradient Focusing (EFGF)

In electric field gradient focusing (EFGF), electric field gradient and an bulk flow are used to enable the net analyte velocity at the focusing point to zero [47]. At the focusing point, the electrophoretic force acting on charged analytes is exactly counteracted by the hydrodynamic force due to bulk fluid flow, while on two sides of focusing point, the imbalance between electrophoretic force and hydrodynamic force causes the analytes to move towards the stationary focusing point. Normally, the bulk flow is constant, and thus a gradient in electrophoretic velocity is required to induce the imbalance between electrophoretic and hydrodynamic forces at two sides of focusing point. In EFGF, electrophoretic velocity gradient is specifically produced by electric field gradient. In comparison with IEF, EFGF can be relatively simple to implement and offers a number of advantages including the use of a single continuous buffer and no limit to analytes with an accessible isoelectric point [48].

Ivory’s group did numerous studies on EFGF focusing of proteins. They created the first-generation apparatus to demonstrate that EFGF has the ability of concentrating and separating proteins [49]. Then, they improved their device to separate and focus charged proteins using the EFGF method by setting a constant electroosmotic flow velocity against step-changing electrophoretic velocity [50]. The device could manipulate electrophoretic and electroosmotic velocities independently. The essential difference between this device and other devices for EFGF was the injection of electric current at discrete intersections in the channel, rather than continuously along the length of a membrane-bound separation channel.

EFGF has been both theoretically and experimentally investigated by Lee’s group. Tolley et al. [51] did a theoretical simulation of EFGF. Their results showed that high peak capacity (over 10,000) is theoretically possible. Humble et al. [52] demonstrated the use of an ionically conductive polymer to produce the required electric field gradient inside the separation channel, which retains large analytes (e.g., proteins), but permits the transport of small buffer ions. This method was shown to be able to concentrate proteins ~10,000 fold within 40 min. A major drawback of this system is that it is difficult to find the optimal operating conditions for the electric field against the hydrodynamic flow due to the lack of field homogeneity. The system also suffers a problem of mass dispersion, leading to the degradation of resolution and sensitivity.

Liu et al. [53] used a novel fabrication method to integrate a conductive membrane into an EFGF device. They found that membrane properties such as conductivity and ion transport could change the electric field gradient produced in the separation channel, which greatly affects the behavior of the EFGF device. A green fluorescent protein was concentrated up to 4000-fold using this EFGF device. Using phase changing sacrificial layers, Kelly et al. [54] also developed an approach to integrate ionically conductive membranes into microfluidic EFGF device. The membrane-based microchips were demonstrated to be able to concentrate fluorescently labelled peptides more than 150-fold and protein samples more than 10,000-fold.

Sun et al. [55] used a bilinear electric field gradient instead of a linear electric field gradient for EFGF to improve the peak capacity. The EFGF induced by a linear electric field gradient could only achieve 20 static peak capacity in a 4 cm long channel, while the bilinear electric field gradient could improve it to a dynamic peak capacity of 150. In addition, they also used a phosphate buffer containing a salt with high mobility ions instead of Tris-HCl buffer to improve the stability of current and reproducibility [56]. A bilinear electric field gradient produced much narrower focusing bands, with a concentration enhancement of 14,000-fold for protein samples. Ansell et al. [57] reported an on-column detection of native, uncolored proteins by fluorescence quenching with EFGF. However, the technique of visualization is only applicable to proteins that absorb at a specific wavelength of 254 nm.

### 3.3. Temperature Gradient Focusing (TGF)

Temperature gradient focusing (TGF) was invented by Ross and Locascio [58] to concentrate and separate charged analytes by balancing the electrophoretic motion of analytes against the bulk flow of solution. The electrophoretic velocity gradient is established by a temperature gradient along the channel with an appropriate buffer. The analytes can then be focused at a stagnant point where the sum of their electrophoretic and bulk velocities is zero. The technique was demonstrated for a variety of analytes, including fluorescent dyes, amino acids, DNA, proteins, and particles (polystyrene) with a concentration enhancement of more than 1000-fold.

**Figure 5:**
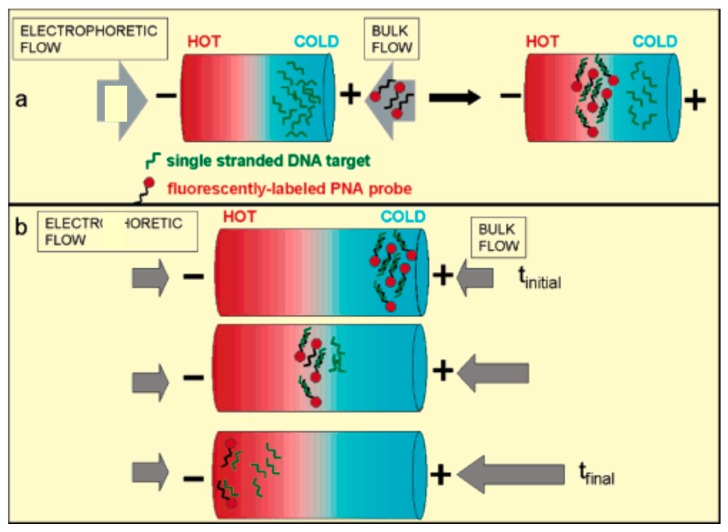
Principle of the two-step temperature gradient focusing (TGF) assays. (**a**) The first-step assay is stationary, and the ssDNA targets are first focused with TGF and then the PNA probe is introduced to the focused DNA with the bulk flow. (**b**) The second-step assay is dynamic. The DNA/PNA hybrid is first focused with TGF to the cold side and then the bulk flow is adjusted to move the analyte against the temperature gradient so that its melting temperature can be determined by observation of the change of fluorescent intensity with temperature. Reprint from Ref. [59].

Balss et al. [59] developed a two-step DNA hybridization assay with TGF (Figure 5). In the first step, ssDNA targets were spatially focused by TGF within a capillary, then peptide nucleic acid (PNA) was transported to the focused DNA by the bulk flow and react with the focused DNA. In the second assay, the PNA/DNA duplexes was first focused with TGF at the cold side of temperature gradient, then as the focused band moved to higher temperature by the flow, the duplexes thermally denatured, and the melting point was identified by a decrease in the intensity of band. The TGF for microfluidic DNA assays has three significant advantages: firstly, targets can be preconcentrated before an assay; secondly, targets can be focused at a stationary point, which facilitates the reaction of targets with probes; thirdly, the target-probe binding partners can be concentrated and spatially separated for sensitive detection. Then the same group [60] further combined micellar electrokinetic chromatography (MEKC) with TGF to concentrate and separate neutral and ionic hydrophobic analytes. This method, which is called micellar affinity gradient focusing (MAGF), is based on a combination of the analytes’ electrophoretic mobility and affinity for their partitioning into an ionic micelle phase. A concentration enrichment of 27-fold for anthracene was achieved within 30 s of performing MAGF. After that, they also demonstrated concentration and separation of chiral compounds by TGF [61]. Different enantiomers of an analyte were focused by TGF at different positions along a microchannel or capillary. Such chirality-induced separations are due to the addition of a chiral selector to the buffer. The chiral selector interacts preferentially with one enantiomer, which shifts its focusing location so that it is separated from the other enantiomer. More than 1000-fold concentration enhancement was reported with the use of chiral TGF. In comparison with capillary electrophoresis, this chiral TGF can achieve a high-resolution separation in much shorter microchannels, and thus is more inherently suitable to be integrated into microfluidic devices for chiral analyses of drugs. However, further efforts should be made to identify low-pH TGF buffers because chiral separation of many drug compounds (such as basic pharmaceuticals) is only feasible at low pH.

One drawback of TGF is the limited peak capacity, and only few analyte peaks (~2–3) can be focused and separated at one time. To circumvent such drawback, Hoebel et al. [62] reported a modified version of the TGF in which the bulk flow rate changes over time. This strategy allows a large number of analytes to be focused, transported to a fixed detection point and washed to the waste in a sequential order. The authors termed this technique the scanning TGF, which enables a more controllable analysis since the scanning time and scanning rate can be actively controlled. More significantly, scanning TGF can be done in simple, short, straight microchannels with no injection structures, which can easily reduce the size of microfluidic device. 

Munson et al. [63] proposed another type of TGF to improve concentration performance. They implemented the TGF with a low conductivity sample buffer and a (relatively) high conductivity separation buffer, field-amplified sample stacking (FASS) also plays roles in sample enrichment in addition to the normal TGF mechanism. This leads to an improvement of normal TGF concentration. The concentration factor for Oregon Green 488 with the modified method was 36-fold higher than the normal TGF. Munson et al. [64] also found that the counterflow necessary for TGF can be used to exclude lower mobility ions, such as serum proteins, when concentrating the higher mobility ions, such as small molecular weight fluorophores. They then further used the counterflow TGF combined with field-amplified sample injection to do the aptamer-based protein assays [65]. 

Normally, the analyte concentration is much lower than buffer ion concentrations. However, when the analyte is concentrated to a significantly high concentration or a low-concentration buffer is used, the analyte concentration cannot be neglected. The nonlinear interactions between analytes and buffer ions can lead to peak shifting and distortion, thereby compromising the detectability and resolution. Lin et al. [66] examined the finite sample effect to elucidate the nonlinear sample-buffer interactions in TGF with theory, simulation and experiment. They derived a generalized Kohlrausch regulating function (KRF) for systems in which electrophoretic mobilities are varying spatially. This generalized KRF greatly simplifies the analysis to a single equation which describes the sample concentration evolution. The peak distortion phenomenon can be predicted by the modified equation, which is consistent with their simulations and experiments.

Dispersion is detrimental to resolution in most focusing techniques, and a clear understanding of dispersion in TGF helps to optimize the technique. Huber and Santiago [67] developed an 1-D model for dispersion in TGF with experimental verification. Their 1-D convection-diffusion equation includes an effective dispersion coefficient for TGF. Dispersion phenomenon can be well explained at low Peclet numbers and relatively low applied electric fields. However, at higher electric field strengths, Joule heating becomes significant and causes spanwise temperature gradients. In this situation, Taylor dispersion analysis fails and ballistic dispersion dominates. Huber and Santiago [68] modified the dispersion model to take into account both molecular diffusion and advective dispersion across all dispersion regimes, from pure diffusion to Taylor dispersion to pure advection. The model was validated by the initial decrease and subsequent increase in peak widths with the increment of electric field strength during the TGF experiments.

Becker et al. [69] applied the TGF to free-flow electrophoresis in microfluidic devices to achieve additional focusing, but only a focusing factor of two fold was obtained for proteins. However, the temperature gradient here is perpendicular to the flow direction which is different from previous reported TGF.

TGF is also a technique for concentration and separation of a mixture of analytes simultaneously. However, one major drawback prevents it from being integrated to portable microfluidic devices. In most studies using TGF, the required temperature gradient is induced by external heating/cooling units, such as electric heating/cooling or water bath. Furthermore, materials with high thermal conductivity (copper blocks) are normally required to be fixed to the capillary device for setting up the temperature gradient. These heating/cooling units as well as copper blocks are usually quite bulky, which makes the device very bulky and thus unsuitable for portable applications. In addition, the use of external heating and cooling equipment requires a significant amount of energy.

Joule heating describes the conversion of electric energy into heat when applying an electric field across finitely conductive media such as electrolyte solutions. In capillary electrophoresis, the presence of such heat will increase the buffer temperature and lead to temperature gradients along both radial and axial directions in the buffer solution, and eventually dissipate into surroundings through the capillary walls. The temperature-dependent buffer and solute properties, including dielectric permittivity, viscosity and diffusivity, would change because of the increasing buffer temperature and the induced temperature gradient. Eventually, the change in these properties would affect the electrophoretic transport of solutes and electroosmosis of buffer solution. In capillary electrophoretic separation, Joule heating usually causes negative effects, such as sample band dispersion or peak broadening, thereby dreading the separation efficiency and analysis resolution. In addition, substantial temperature increment may result in the decomposition of thermally labile samples and even the formation of vapor bubbles. However, Joule heating can be positively used under some circumstances, such as to control the thermal environments in microfluidic devices. Enhancement of heat transfer by Joule heating induced thermal convection was analytically examined using idealized thermal boundary conditions [70,71,72]. A normalized source term is introduced to represent the ratio of Joule heating to surface heat flux by Horiuchi et al. [73,74], in their thermal analysis of pure electroosmotic flows and mixed electroosmosis/pressure-driven flows. Studies of Joule heating effects were further extended to electroosmotic flow and mass species transport under steady-state and spatiotemporal conditions [75,76,77].

One constructive proposal is using Joule heating to induce temperature gradient required for TGF, in lieu of the conventional use of external heating/cooling equipment. The advantages include less power consumption, simple and compact design, and more portable device without need of bulky external heating units. Such idea was first demonstrated by Kim et al. [78] experimentally. The temperature gradient for TGF in their experiments is established by Joule heating effects coupled with the variable channel width. Much less power and energy per analysis is consumed for the Joule heating (0.08 W) compared to the external heating/cooling (6.4 W) to produce the similar temperature gradient focusing. A mixture of two sample analytes was concentrated and separated within 10 min. A quasi-1D numerical model was proposed by Sommer et al. [79] to describe the resulting temperature, velocity, and concentration profiles in the microchannel for Joule heating induced TGF. Their numerical results showed good agreement with the experimental data presented in their previous work [78]. The effects of varying channel geometries and adjusting the temperature of substrate on focusing performance were studied with the numerical model.

**Figure 6:**
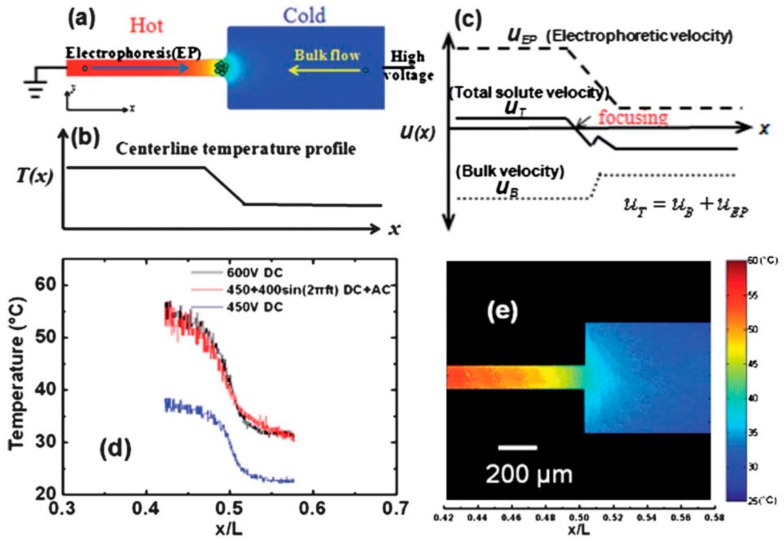
Principle of Joule heating induced TGF in a microfluidic sudden expansion under a combined AC/DC electric field. (**a**) Joule heating in the microfluidic expansion produces a temperature gradient around the junction region connecting narrow and wide channels; (**b**) temperature distribution along the centerline of microchannel (axial direction); (**c**) velocity profiles of sample solute along the centerline of microchannel due to pure electrophoresis (dashed line), pure bulk flow (dotted line), a combination of electrophoresis and bulk flow (solid line); (**d**) temperature distributions along the centerline of a 10 mm long polydimethylsiloxane (PDMS) microchannel for three different applied voltages: 450 V DC, 450 V DC + 400 sin(2π*ft*) V AC and 600 V DC, measured with the rhodamine B thermometry; (**e**) temperature contour in the microfluidic expansion for an applied voltage of 450 V DC + 400 sin(2*pft*) V AC, measured in 180 mM Tris–borate buffer solution. Reprint from Ref. [82].

Tang and Yang [80] developed a 3D numerical model for describing the Joule heating induced TGF. The numerical model consists of a set of governing equations to describe the temperature gradient and concentration profiles. The thermophysical and electrical properties including the liquid dielectric constant, viscosity, electric conductivity and mobility are all temperature-dependent. The numerical simulations showed good agreement with the experimental results presented by Ross and Locascio [58]. Later, Yang’s group invested tremendous effect in experiments to use TGF for analyte concentration. A microchannel with a sudden expansion was designed to produce temperature gradient for the TGF focusing of sample solute [81], and a systematic study showed that higher applied voltage, higher buffer concentration or larger channel width ratio can improve the TGF concentration. The technique was further improved with the use of AC/DC combined electric fields [82,83], see Figure 6. The proposed AC/DC combined field technique led to more than 2500-fold concentration enhancement of Fluorescein–Na solute, which is unprecedentedly high for the Joule heating induced TGF technique. Recently, the group also applied the AC/DC combined field driven TGF to concentrate DNA, and a rapid concentration enhancement of DNA was achieved with 480 folds in 40 s [84].

## 4. Other Microfluidic Concentration Methods

### 4.1. Electrokinetic Trapping (EKT)

**Figure 7:**
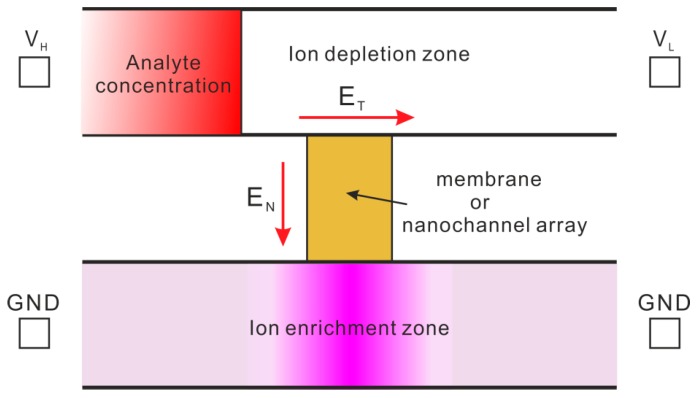
Schematic illustration of a typical electrokinetic trapping (EKT) concentration device. The device consists of two microchannels that are connected by a nanopore membrane or nanochannel array. Under the effect of applied voltages, ion concentration polarization is developed at the membrane-microchannel interfaces, giving rise to the development of an ion enrichment zone inside the lower channel and an ion depletion zone inside the higher channel. The ion depletion zone does not allow the passage of any charged species, thereby leading to the accumulation of charged analytes in front of the ion depletion zone.

Electrokinetic trapping (EKT) is a result of ion concentration polarization which is a universal phenomenon near ion selective membranes or microchannel-nanochannel junctions. Figure 7 illustrates a typical EKT sample concentration system by ion concentration polarization. The system consists of two microchannels that are connected by an ion selective membrane or nanochannel array. During operation, two ends of the lower channel are electrically grounded (GND), and two ends of the higher channel are provided with voltages, *V*_H_ and *V*_L_, and *V*_H_ is maintained to be larger than *V*_L_. Then, the electric fields inside the upper microchannel and the membrane, *E*_T_ and *E*_N_, are induced, as is shown in the figure. Under the effect of *E*_N_, only ions with polarity that is opposite to the polarity of membrane surface charge can penetrate through the membrane (In most cases, the membrane has negative surface charges, so only cations can pass through the membrane). Such selective transport of ions through membrane at equilibrium leads to the formation of an ion enrichment zone inside the lower microchannel while an ion depletion zone in the higher microchannel. This is characteristic of ion concentration polarization [85,86]. Since ion depletion zone allows no existence of charged species, the left boundary of ion depletion zone must prevent the passage of any charged species from the analyte zone to ion depletion zone. Therefore, the charged analyte from analyte zone would be trapped near the left boundary of the ion depletion zone (the unshaded zone inside the higher microchannel), and gradually the concentration of charged analyte is enhanced [85,87]. The stability of EKT is dependent on various factors such as pore size, charge density of the nanopores membrane, and counteracting flow which one has to take into account for fabricating a viable concentration device.

EKT method was first reported by Dai et al. [88] who obtained a concentration enhancement of DNA more than 100 fold using an ion-selective membrane. Hahn et al. [89] developed a field-amplified EKT by using poly(ethylene terephthalate) (PET) membranes. The technique was shown to be able to concentrate DNA molecules from a very large volume. Their findings suggest that the field-amplified EKT performs much better than conventional EKT, and also the increasing pore size of membrane has a negative impact on analyte concentration.

Wang et al. [90] improved the EKT technique to achieve a million-fold concentration enhancement of proteins and peptides within several hours with a nanofluidic filter. The nanofluidic filter functions as an ion-selective membrane to generate an ion-depletion region, which was used to trap and concentrate biomolecules. The nanofluidic filter in the device is one single nanochannel with well-defined dimensions (40 nm in depth), which was fabricated with standard soft lithography and deep reactive ion etching on silicon wafers. Unlike membranes, nanofluidic channels do not swell or shrink in response to the change of ionic strength, temperature, or other environmental conditions. This makes the device more controllable than the use of ion-selective membrane. Nevertheless, nanochannels require the use of sophisticated clean room fabrication techniques, and thus are more expensive than commercially-available membranes. Wang and Han [91] utilized the nanochannel-based EKT as a preconcentration technique to enhance immunoassay detection sensitivity and binding kinetics. Their EKT devices have multiple well-defined nanochannels to induced ion depletion zone. With 30 min preconcentration, more than 500-fold enhancement of immunoassay sensitivity was obtained.

For a more systematic review of the EKT technique, we recommend the works [85,87,92]. Here we would only review the most recent development of the technique. Instead of using the conventional two-channel design, Ko et al. [93] simplified the device design of EKT to a single channel. The robustness of EKT was demonstrated with various material combinations such as polydimethylsiloxane (PDMS) channel-glass substrate and silicon channel-PDMS substrate. Because of excellent biocompatibility of PDMS, there are increasing uses of PDMS as channel materials for EKT concentration of biomolecules [93,94,95,96,97,98]. Recently, paper-based channels were also used in the EKT concentration device [99,100]. Paper-based materials would make the device more disposable and cheaper. Apart from the channel material, the membrane material is also important for EKT device, since it determines the ion concentration polarization that is essential for concentrating analytes. Recent development focuses on the use of Nafion membrane [93,94,95,96,97,98,99,100]. Such a trend is mainly due to two reasons: the fabrication process of Nafion membrane EKT device is simple and easy; the surface charge density of Nafion membrane is significantly higher than that of bare silicon nanochannels or other membranes, and much higher concentration factors could be achieved. Further improvement in fabrication of EKT device was reported by Phan et al. [101] who developed a robust method using an off-the-shelf Nafion membrane. It was demonstrated that EKT device fabricated with the off-the-shelf Nafion membrane led to a stronger ion-concentration-polarization repulsion force than that fabricated with Nafion resin for the same channel dimension and input parameters. The new device thus could greatly enhance the separation efficiency. In addition, EKT device with the off-the-shelf Nafion membrane has better mechanical stability and thus a longer lifetime compared to the existing method.

### 4.2. Dielectrophoretic Trapping (DEPT)

**Figure 8:**
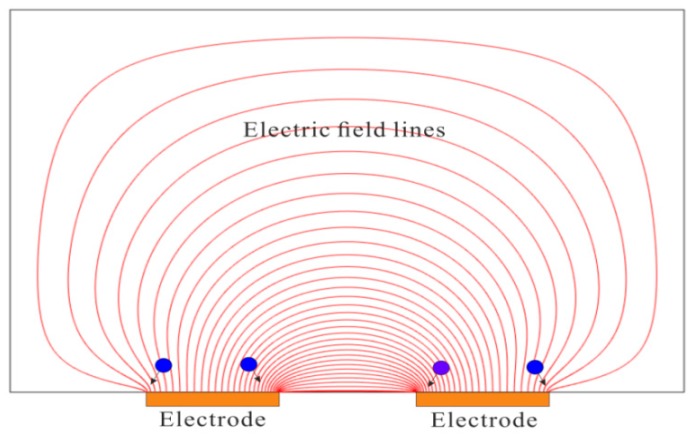
Schematics of dielectrophoretic (DEP) trapping of particles over edges of coplanar electrodes. The density of electric field lines determines the electric field strength, and it is clear that the electric field strength is the highest over the edges of electrodes. Under the effect of positive DEP force, particles would move from the region of low electric field strength (center of electrode) to the region of high electric field strength (edges of electrode).

When electrically polarizable particles/macromolecules are subject to in a nonuniform electric field, the particles experience dielectrophoretic (DEP) force [102], which can be used to trap the particles. Asbury and van den Engh [103] showed the concentration of DNA in nonuniform AC electric fields by the DEP force. Their design consists of an array of coplanar electrodes which are energized with AC electric field. The electric field is extremely condensed near the edges of electrodes, and hence DNA molecules tend to move to the edges of electrodes because of positive DEP force. More DNA molecules are trapped over the edges of electrodes, leading to the enhancement of DNA concentration. The dielectrophoretic trapping (DEPT) mechanism in this case is graphically illustrated in Figure 8. Application of a voltage between two coplanar electrodes generates an electric field which is most condensed near the edges of electrodes. Particles experiencing positive DEP force move from the region of low electric field strength to the region of high electric field strength. Similar electrode design was also used to concentrate listeria cells [104]. To make DEPT more affordable and commonplace, Park et al. [105] directly used the interdigitated electrodes on a printed circuit board (PCB) for DEPT of HeLa cells and polystyrene particles. Their method does not requires use of sophisticated microfabrication processes, and greatly reduces the cost of device fabrication. More significantly, well-established processing methods in PCB industry allow mass production of their DEPT device. DEPT uses the dielectrophoretic force to collect particles on electrodes. However, the dielectrophoretic force is only present in the very proximity of electrodes. Therefore, DEPT is only able to capture particles that are very close to electrodes, and the concentration enhancement of particles is limited. To overcome such limitation, various micromixer configurations, including the slanted groove, staggered herringbone, and herringbone mixers were proposed to circulate the sample, increasing the probability for the particles to reach the electrodes for trapping [106,107].

Du et al. [108] designed a quadrupole electrode platform to induce DNA molecules focusing with the collaborative effects of long-range AC electroosmotic flow and short-range DEP force. The AC electroosmotic flow convects DNA molecules to the regions near electrodes, and then DEP force further traps these molecules towards electrodes. Several tens of enhancement for pico-molarity DNA solutions were achieved without any continuous sample supply. They further proposed a double half-quadrupole electrode system to induce a head-on AC electroosmotic streaming for focusing and trapping DNA molecules. The observed phenomenon is a combined result of the formation of two prefocused DNA jets by AC electroosmotic streaming, dipole-induced attraction between focused DNA molecules, and dielectrophoretic trap near electrodes [109]. In addition to DNA molecules, they also employed the same design for submicrometer particles. The results showed that the trapping of particles is strongly dependent on the AC frequency and particle size. For large particles (e.g., 0.92 µm) and high frequency (20 MHz), DEP plays a significant role, while for small particles (e.g., 0.1 µm) and low frequency (1 kHz), AC electroosmosis plays a more important role than DEP. 

With numerical modelling, Loucaides et al. [110] investigated the effect of initial particle concentration on the DEP trapping in an array of coplanar parallel electrodes. The initial concentration was found to have significant impact on the initial rate and steady state of particle collection and the study also showed that AC electroosmosis enhances both of them. They further modelled the trapping of DNA in a same electrode design with consideration of steric effects in the suspension motion [111]. The results from the steric model are more realistic than those from the non-steric model.

**Figure 9:**
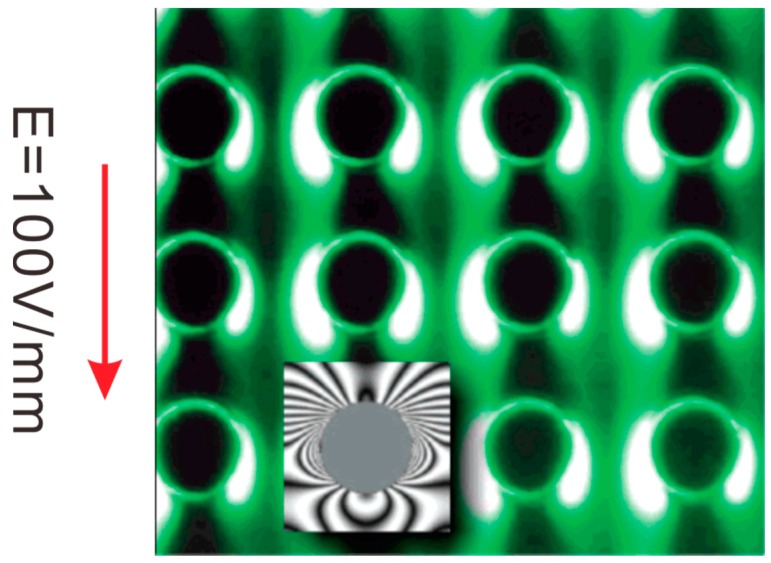
Dielectrophoretic trapping (DEPT) of fluorescent particles by an array of insulating pillars. The pillars are 33 μm in diameter and with a center-to-center separation of 63 μm. The inset picture shows the isopotential lines around one pillar from numerical simulation. Dielectrophoretic traps are defined by regions with isopotential lines bending back onto a pillar surface. Reprint from [112].

The creation of nonuniform electric fields is essential for DEPT. Most studies in the literature, such as those reviewed above, create nonuniform electric fields by specific arrangement of electrodes. Such method is most widely used, but usually requires complicated electrode design and also faces the problem of electrode fouling and degradation. An alternative way of producing nonuniform electric fields is by embedded obstacles such as a specifically arranged array of insulators. Insulators suffer much less from fouling and degradation. In addition, insulators made of polymer-based materials can be mass produced with standard microfabrication processes, giving rise to cheap and large-volume devices. Such concept of insulator-based DEPT was first presented by Cummings and Singh [112] who fabricated a microfluidic device with an array of insulating pillars in a microchannel. One electrode in the microchannel inlet reservoir and the other electrode in the microchannel outlet reservoir are supplied with a DC voltage to set up an electric field along the microchannel. Then nonuniform electric field strength is induced due to the geometric confinement between insulating pillars. The DEPT of 200 nm fluorescent polystyrene particles were successfully demonstrated, as is shown in Figure 9. Chou et al. [113] demonstrated insulator-based DEPT of DNA molecules using AC electric fields. Lapizco-Encinas et al. [114] investigated the selective DEPT of polystyrene particles, live *E. coli*, and dead *E. coli* in an array of insulating micropillars under DC electric fields. The same group reported the selective DEPT of four different types of live bacterial cells, including the Gram-negative Escherichia coli and the Gram-positive Bacillus subtilis, Bacillus cereus and Bacillus megaterium [115]. Mela et al. [116] elaborated the effect of zeta potential of device material on insulator-based DEPT, and found that Zeonor 1060R substrates require lower trapping voltage thresholds as compared to glass substrates. This is due to the fact that the Zeonor substrate has a zeta potential that is lower than the glass substrate under a same physicochemical condition.

In addition to using arrays of insulating pillars, other types of configurations were also proposed to induce nonuniform electric fields required for insulator-based DEPT. Chen et al. [117] reported a rapid concentration of nanoparticles with DEPT induced by DC electric fields. The nonuniform electric field was produced by a series of PDMS microchannels with a tree-like arrangement. Lewpiriyawong et al. [118] utilized a microfluidic constriction to generate nonuniform electric fields for insulator-based DEPT of particles and cells. Zhao et al. [119] recently proposed a novel design of using asymmetric orifices on opposite channel walls to generate nonuniform electric fields. Such design was shown to greatly reduce the magnitude of voltages required for insulator-based DEPT. 

Hawkins and Kirby [120] theoretically investigated the electrothermal flow in polymeric, insulator-based DEPT systems with DC-offset, AC electric fields at finite thermal Peclet number. It was shown that electrothermal effects always enhance dielectrophoretic trapping of particles. Sridharan et al. [121] presented the first experimental study of electrothermal effects on insulator-based DEPT, and the results are found in line with the previous theoretical predictions.

### 4.3. Immunocapture Based Trapping (ICBT) 

**Figure 10:**
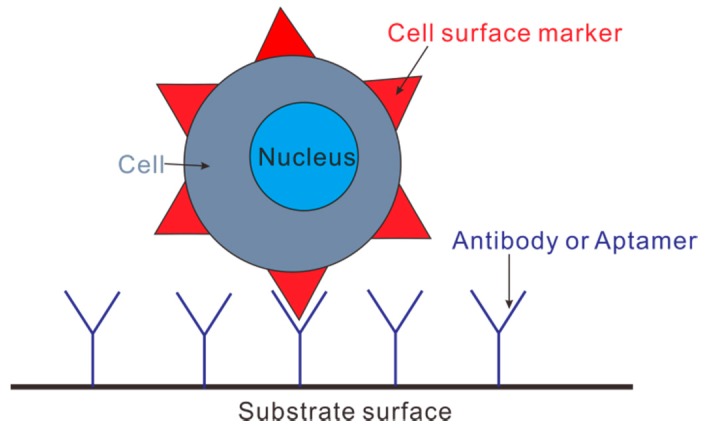
Schematic principle of immunocapture of a circulation tumor cell (CTC). An antibody or aptamer chemically immobilized on the substrate surface can be specifically linked with cell surface markers extruding from the cell membrane.

Immunocapture is a technique for increasing the concentration of biological cells. It is analogous to affinity chromatography in principle. The central idea of immunocapture is that cells are captured by a specific interaction between cell surface and substrate [122]. Take immunecapture of a circulating tumor cell (CTC) as an example (Figure 10), one type of cell surface marker (e.g., protein or receptor) extrudes from the cell membrane. An antibody or aptamer, which is chemically linked to substrate surface by the so-called immunochemistry, specifically interacts *only* with that type of cell surface marker. This process leads to immobilization of cells on the substrate, and thus the concentration enhancement of CTCs.

Immunocapture-based trapping (ICBT) can collect CTCs directly from whole blood samples. This means that blood sample does not need any pretreatment, thereby simplifying the procedure greatly. ICBT has two modes of operation: affinity-based or depletion-based cell concentration. Affinity-based concentration accumulates CTCs from the whole blood sample [123,124,125], while depletion-based concentration [126,127,128] removes undesired components of blood, and only CTCs are left behind. Generally, ICBT is an efficient method of CTC isolation, on condition that cancer cells have a specific type of surface marker, and there is also an antibody/aptamer which only binds with that type of surface marker. In addition to concentrating CTCs, ICBT was also used for preconcentration of proteins and HIV subtypes in biological samples to facilitate subsequent detection [129,130].

In practical implementation, ICBT concentration has several typical device designs: micropillar arrays [123,125,131], micro-/nanostructured surfaces [132,133,134], micromixers [135,136], and microbeads [137,138,139,140,141]. Micropillar arrays use geometric obstacles to increase the surface area to volume ratio, thereby enhancing the frequency of CTC-antibody interaction and then the resultant cell capture. Micro-/Nanostructured surfaces use micro/nano fabrication technology to create roughness on flat substrates; this equivalently increases the surface area to volume ratio and thus the probability of cell-antibody collision and the corresponding cell capture. Micromixers use microfabricated ridges to create flow circulations in the device. CTCs in the circulating flow interact with the walls of the device which are the coated with a CTC-specific antibody or aptamer for cell capturing. Microbeads are usually magnetic and coated with an antibody which tends to bind with a specific cell surface marker. When placed into blood samples, microbeads bind specifically with CTCs to result in cell capture. Finally, the application of a magnetic field across microbeads releases CTCs for later analysis. Among these four device designs, microbeads are the most promising for clinical use because the only ICBT device approved by Food and Drug Administration (FDA) at present (the Veridex CellSearch) uses microbeads [142,143,144].

The ICBT technique has two major drawbacks [122]: (i) A good immunocapture device requires a cell surface marker which has a uniform expression for all CTCs and an antibody or aptamer which is 100% specific to the CTC, namely without any non-specific cell binding. However, the cell surface marker expression in practice varies from CTC to CTC, and the target antibody can be too specific (losing the chance of binding with altered markers), or not specific enough (producing more non-specific binding). (ii) For effective immunocapture, one has to know a priori the cell surface markers expressed by CTCs. Yet, this is rarely the case in practice. For example, a pan-cancer CTC cell surface marker has been shown to be downregulated by some cancer cells in order to elude detection by the immune system, and to enhance their mobility in the body. The risk of missing entire populations of CTCs that do not express the target cell surface marker is problematic when attempting to identify the most dangerous CTCs. 

**Figure 11:**
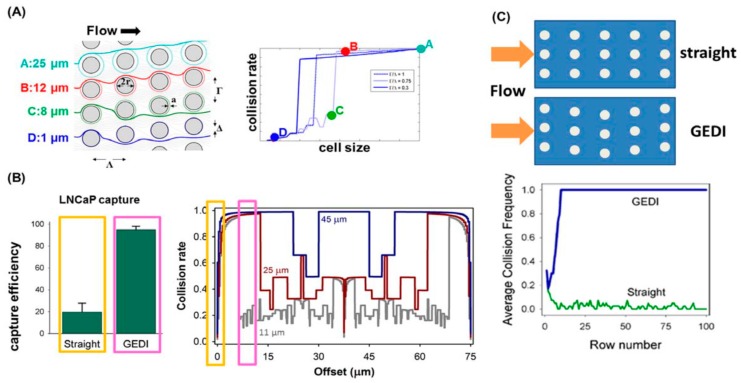
A typical geometrically enhanced differential immunocapture (GEDI) microfluidic device for cell capture. (**A**) Left: Top view of micropillar array. Δ is pillar offset; Λ is pillar separation in the direction of bulk flow; Γ is the pillar separation in the direction perpendicular to bulk flow; 2*r* is the pillar diameter. Light gray lines represent the fluid flow streamlines. Color lines denote trajectories of cells of various sizes. Right: The cell-wall collision rate varies with the cell size and the offset parameter of the array; GEDI prefers size-dependent collision rates introduced by a nonzero offset parameter. The solid lines are the results predicted for the flow geometry at left; four colored dots are corresponding results of four specific cell sizes. The dotted and dashed lines are results predicted for other two offset parameters; (**B**) the comparison of measured LNCaP capture efficiency on J591-functionalized devices for GEDI (7-mm offset) and straight (no offset) geometries (both geometries have the same surface-area-to-volume ratio). Right figure shows the simulated collision rates in these geometries. Straight arrays or arrays with small offsets (yellow boxes) induce low capture efficiency (left) and size independence (right). In contrast, arrays with large offsets (magenta boxes) induce high capture efficiency (left) and size dependence (right); (**C**) the collision frequency in straight (GEDI) array decreases (increases) as the sample flows through the device. Reprint from Ref. [148].

Some recent efforts have been made to improve the performance of classic immunocapture. In conjunction with dielectrophoresis, immunocapture was shown to be able to maximize the capture of target rare cells, while minimizing the capture of contaminating cells [104,145,146]. Another important development by Kirby and his colleagues is the so-called geometrically enhanced differential immunocapture (GEDI) microfluidic devices that capture rare cells from blood or cell suspensions [125,131,147,148]. Figure 11 decribes the principle of the GEDI capture in detail. GEDI combines the cancer-specific antibody immunocapture with micropillar array to enhance the probability of the collision of larger cells (e.g., CTCs) with the substrate, while minimize the probability of the collision of smaller contaminating cells (e.g., leukocytes) with the substrate. The GEDI platform has been successfully used by the authors for concentrating pancreatic cancer cells [131], prostate cancer cells [148,149], breast and gastric cancer cells [150]. Very recently, Sarioglu et al. [151] developed a microfluidic device (the cluster-chip) to capture CTC clusters instead of single CTCs from unpretreated blood without the need of tumor-specific markers. CTC clusters were physically captured by geometric obstructions with low-shear liquid flow. For 30 to 40 percent of patients with metastatic breast or prostate cancer or with melanoma, the device was able to identify CTC clusters. The ability to capture CTC clusters is much needed for subsequent characterization of their biological properties and roles in metastasis.

### 4.4. Magnetic Beads Assisted Trapping (MBAT)

**Figure 12:**
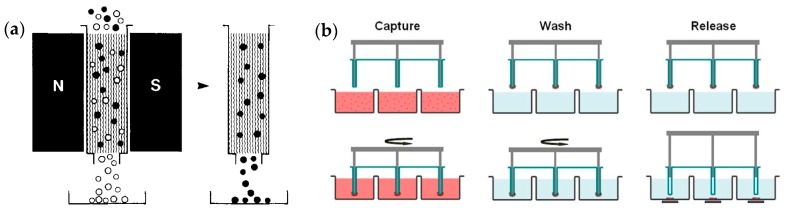
Two typical systems of magnetic beads assisted trapping and releasing of cells. (**a**) Column-based system. Left: Under the effect of external magnetic field, cells labeled with superparamagnetic nanobeads (black dots) are trapped on the matrix of column, while unlabeled cells (white dots) can go through the column. Right: The trapped cells can be released as soon as the magnetic field is removed. “N” and “S” denotes the north pole and south pole of the magnet, respectively; (**b**) MagSweeper system. Blood samples in the capture wells are labeled with magnetic nanobeads. The magnetic rods covered with a thin layer of plastics sweep through the wells in concentric circular loops to magnetically attract target cells labelled with nanobeads. Loosely attracted unwanted cells are washed off from the magnetic rods with them moving in circular loops. Finally, the magnetic rods are immersed into a new buffer solution and disengage from the plastic covers. Cells labeled with magnetic nanobeads are released under the effect of magnetic fields setup by magnets located under the wells. Figure (**a**) is reprinted from [152], Figure (**b**) is reprinted from [154].

The magnetic beads assisted trapping (MBAT) relies on the magnetic force to trap cells. Figure 12 illustrates two classical systems of MBAT. In the column-based system, cells labeled with superparamagnetic nanobeads (in the order of 100 nm) are retained in the column because of magnetic trapping force, and unlabeled cells are eluted. The removal of external magnetic field would release the labeled cells [152]. Such system has been commercialized for sorting cells (such as Manual MACS by Miltenyi Biotec) with high magnetic field gradient in a column. However, the throughput as well as efficiency of isolating rare cells using such commercial system is usually low because of manual operation and limited magnetic field strength [153]. In the MagSweeper system, magnetic rods covered with plastics are used to magnetically attract target cells conjugated with magnetic nanobeads, and later trapped cells are released under the effect of external magnetic field [154]. Such system is new and only reported recently, and it can use multiple rods to perform parallel analyses, while the trapping efficiency could be compromised by the reduction of magnetic field strength due to plastic covers. In both MBAT systems, preparation of cell sample is similar. There are typically three steps required to labelling cells with nanobeads which are usually superparamagnetic and biotinylated [152]. The cells are firstly labelled with biotinylated antibody, and secondly dyed with a fluorescent avidin conjugate, e.g., fluorescein-steptavidin. Finally, the biotinylated superparamagnetic nanobeads bind with the avidin on the cell surface. Thus, antibody binding cells finally have a fluorescent and a magnetic label. The choice of nanobeads for cell labeling is critical for MBAT, and there has been a wealth of information regarding their preparation, characterization, functionalization and current development, as are reviewed in Refs. [155,156]. 

MBAT has been advancing quite fast in recent years and playing important roles in the study of CTCs. However, the traditional MBAT devices are incapable of meeting the requirements for sophisticated molecular studies. Additionally, the traditional MBAT devices are quite bulky and thus unsuitable for point-of-care applications, and also prone to cross contamination. With the rapid development of micro- and nanofabrication techniques, microfluidics-based MBAT emerges as a new technology to provide more precise and flexible control of the magnetic and hydrodynamic forces experienced by the target cells to improve cell enrichment. Similar to the traditional MBAT system, a microfluidics-based system uses an external magnetic field to capture target cells which are labelled with magnetic nanobeads. Depending on the way in which the target cells are enriched, microfluidics-based MBAT has two working modes [153]: an retaining mode which magnetically traps CTCs on the substrate [157], and a deflecting mode which magnetically drives CTCs to form discretized concentrated streams [158,159]. 

There have been continuous efforts to improve MBAT. Luo et al. [160] developed a new microfluidic system with integrated micromixers and micropumps to achieve “negative selection and enrichment” of CTCs. Under an external magnetic field, leukocytes labelled with anti-human CD45 antibodies-coated magnetic beads were removed, and target cells left behind were effectively enriched. Wang et al. [161] reported the use of a herringbone microstructure in a microfluidic MBAT chip. The introduction of a herringbone microstructure produces two favorable effects: (i) the herringbone microstructure induces vortices which bring CTCs close to herringbone grooves. This increases the possibility of trapping CTCs into herringbone grooves by the magnetic force; (ii) Magnetically trapped CTCs in the herringbone grooves experience significantly smaller shear stress compared to that in the microchannel. This would prevent damaging of CTCs. 

### 4.5. Thermophoretic Trapping (TPT)

**Figure 13:**
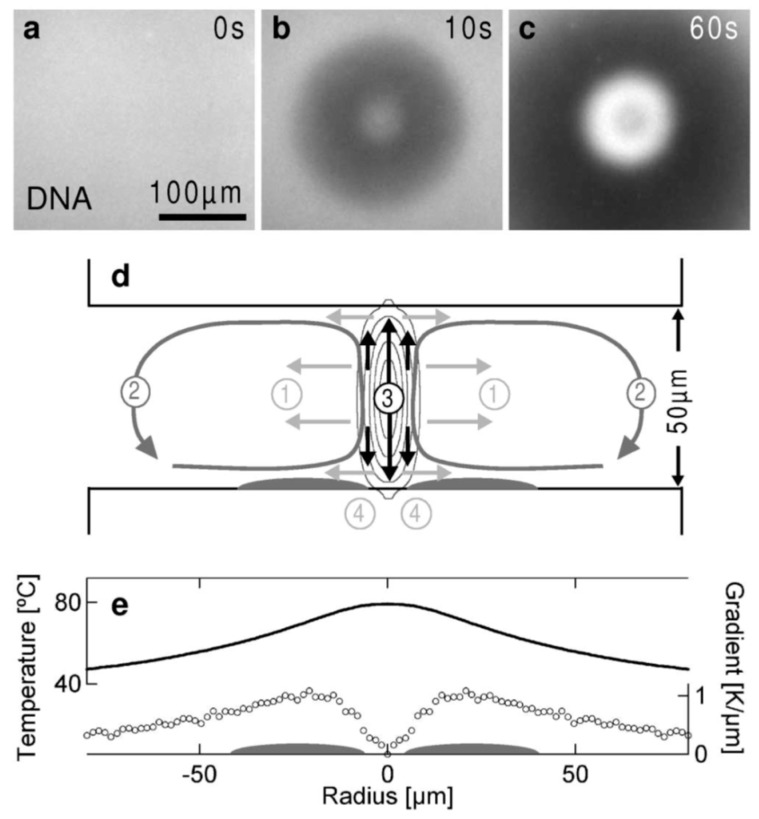
Mechanism of the thermophoretic trapping (TPT) technique for concentrating DNA. (**a**) Initial DNA distribution without any heating; (**b**) fast DNA depletion due to thermophoresis; (**c**) DNA concentration due to the combined effect of thermophoresis and thermal convection; (**d**) the TPT is a synergistic effect of lateral thermophoresis (1)/(4), axial thermophoresis (3) and convection (2); (**e**) the measured lateral temperature distribution around the heating spot. Reprint from Ref. [162].

Thermophoresis describes the movement of molecules or particles in response to a temperature gradient. This phenomenon can be used to achieve the enhancement of analyte concentration. Braun and Libchaber [162] developed the thermophoretic trapping (TPT) technique which utilizes a synergistic effect of thermophoretic depletion and thermal convection to concentrate DNA. The mechanism behind this technique is depicted in Figure 13 which shows the concentration of DNA with laser-induced heating inside a chamber with thickness of 50 µm. At beginning, the dominant thermophoretic force quickly induces a depletion of DNA from the heating spot (Figure 13b), and then thermal convection counteracting the depletion brings DNA molecules towards the heating spot and causes the concentration of DNA (Figure 13c). The concentration of DNA around the hot spot is a result of the interplay between thermophoresis and thermal convection, as sketched in Figure 13d. These combined effects create a stagnant zone on the lower surface of the chamber near the laser heating spot (the gray area in Figure 13d), where accumulation of DNA occurs. More than 1000-fold enhancement of DNA concentration was achieved within 180 s. The measured temperature distribution around the hot spot is shown in Figure 13e, and the ring-shaped concentration zone can be explained by the ring-shaped temperature gradient which triggers the TPT process.

The same group later showed that thermophoretic characteristics can be adjusted by controlling the background temperature. It was shown that the DNA has a thermophoretic concentration at 3 °C, but a thermophoretic depletion at room temperature [163]. They also utilized the TPT to greatly enhance the efficiency of polymerase chain reaction (PCR) process for DNA replication [164]. 

The development of TPT induces some variations of the technique. Instead of using the concurrent thermally-induced convection flow, Duhr and Braun [165] applied an external pressured driven flow to counteract thermophoresis. The technique achieves an efficient concentration enhancement (800-fold) of DNA molecules. In comparison with the original TPT technique, this modified TPT has a well-controlled microfluidic condition. Other than the conventionally-used laser induced heating, Serey et al. explored the use of a photonic crystal resonator to induce localized heating required for TPT [166]. Their experiment showed a similar effect of DNA concentration enhancement.

### 4.6. Other Electrokinetic Methods

There are some other methods of analyte concentration which cannot be classified into the above reviewed categories. Khandurina et al. reported [167] a microfabricated injection valve incorporating a porous membrane structure to concentrate DNA samples. The porous membrane was fabricated in the microchannel with two channels separated from each other and connected by a thin porous silicate layer. Large molecules, such as DNA, accumulate before the membrane due to their hindered transport through the membrane, and the concentration enhancement of 2 orders of magnitude can be achieved within 3 min. Later, the same technique was also used to preconcentrate proteins [168]. Similarly, Kuo et al. [169] developed a micro-CE chip for DNA preconcentration and separation using a normally closed valve which can be activated by pneumatic suction. The negatively charged DNA was blocked and trapped by a nanoscale channel formed by the normally closed valve with anionic surface charges. An approximate 41 times concentration enhancement was obtained after 100 s. However, the normally closed valve brings complexity in design and fabrication of microchips, and also such device is prone to mechanical failure.

Kuo et al. [170] developed an on-column concentration and separation of dsDNA using gradient capillary electrophoresis. To concentration DNA prior to separation, they introduced concentration gradient of polymers along the capillary, such as PEO (polyethylene oxide) and EtBr (ethidium bromide). The concentration of DNA is mainly due to the sieving and the change in viscosity as DNA migrates from sample zone to the polymer solution. Several ten-fold sensitivity improvements were demonstrated in the separation of DNA mixtures. Park and Swerdlow [171] also developed an on-line DNA sample concentration method for enhancing the sensitivity of capillary electrophoresis. The method allows an effective capture of DNA fragments from a flowing sample stream which is appropriately opposed by a strong electric field. The captured DNA fragments form a concentrated band by simply turning off the electric field. In addition, an even more concentrated band of DNA can be recovered when reversing the polarity of the electric field during the elution process.

**Figure 14:**
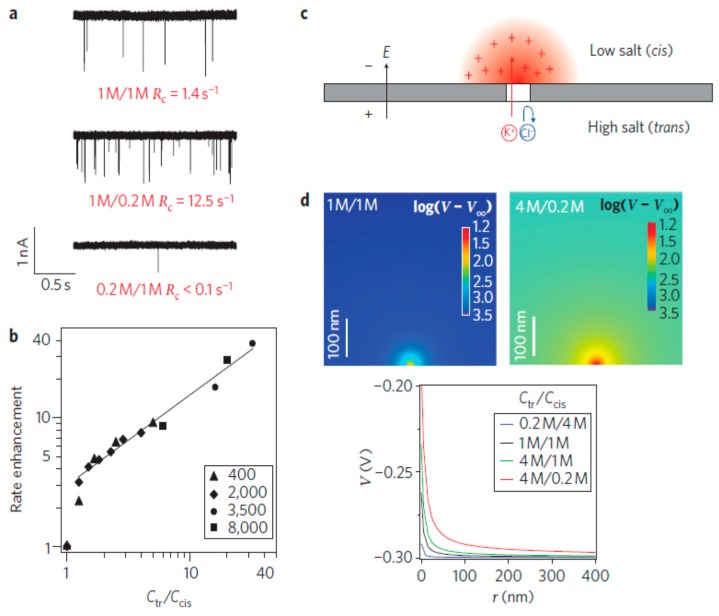
Intensified DNA molecules focusing into a solid-sate 3.5-nm nanoscale pore by salt gradients. (**a**) Measured continuous traces with 400 bp DNA for different KCl concentration (*C_tr_*/*C_cis_*) ratios. *R*_c_ value in each case is the average capture rate; (**b**) enhancement of DNA capture rate with *C_tr_*/*C_cis_* for four different DNA lengths; (**c**) schematic showing of the selective transport of cations (K^+^) from *trans* to *cis* for the case of *C_tr_*/*C_cis_* > 1, leading to a local accumulation of cations near the pore on the *cis* side (reddish area), which enhances capture rate; (**d**) numerical electric potential distributions around the pore at the *cis* side, for both symmetric (top left) and asymmetric (top right) KCl concentrations. The bottom center of each image is where the pore is located. Reprint from Ref. [172].

Wanunu et al. [172] studied the focusing of DNA into solid-sate nanoscale pores with a salt gradient (Figure 14). More DNA molecules are captured when ionic gradients are established across the pore and the capture rate is increased as increasing the salt gradients (Figure 14a,b). The nanopore is negatively charged, and thus only cations can pass through the nanopore from *trans* to *cis*. Such selective transport of cations leads to the formation of a zone on the *cis* side (reddish area in Figure 14c), which mainly contains cations. The cation concentration inside this zone is decreasing as the distance is away from the pore, which induces an enhancement of the local electric field around the pore (Figure 14d). Therefore, more molecules are electrophoretically focused into the pore, raising the possibility of using nanopores to analyze unamplified DNA samples. High throughput detection of picomolar DNA concentrations was demonstrated with a 20-fold salt gradient. Instead of using a salt gradient, Paik et al. [173] exploited the use of electrically-gated nanopores to achieve a flexible control of DNA focusing. It was demonstrated that the DNA capture rate could be adjusted over 3 orders of magnitude with gate voltage less than 1 V. Such efficient control of DNA focusing arises from the appropriate balance between electrophoresis and electroosmosis.

Dhopeshwarkar et al. [174] developed an electrokinetic focusing method using bipolar electrode (BPE) in a microchannel, where the electrophoretic velocity of anions is balanced by the electroosmotic velocity due to the control of local electric filed by BPE. Theoretical description and numerical simulations were also reported to shed light on the complex local interplay among electrokinetics, hydrodynamics, and electrochemistry involved [175]. Perdue et al. [176] extended the method by using discontinuous BPEs to show that concentration enrichment is coincident with the onset of faradaic electrochemistry at the BPE. About 70-fold concentration enrichment was achieved within 160 s. 

Dghighi and Li [177] proposed an electrokinetic method for concentrating DNA molecules in a straight microchannel with two closed ends. The concentration is a joint effect of electroosmotic flow, electrophoresis and the flow induced by back pressure. The concentration process and the transport of DNA molecules feature a flexible control by the manipulation of the voltages via three electrodes. 91-fold concentration enrichment can be obtained within 115 s. Separation of the focused DNA with different mobilities also can be realized while transporting them downstream along the microchannel.

## 5. Summary and Conclusions

Lab-on-a-chip can integrate various laboratory and analytical protocols ranging from mixing, pumping to concentration, separation and detection etc. into a single microchip. It is an emerging microfluidic technology which is believed to have the great potential to revolutionize chemical and biological analyses. However, Lab-on-a-chip devices normally suffer from low analysis sensitivity due to the inherently poor detection limits in microchannels. To improve the analysis sensitivity of lab-on-a-chip devices has prompted the need of on-chip preconcentration of analytes. A variety of microfluidic techniques have been developed for analytes concentration. This paper reviews these techniques and focuses on discussing their physical principles and advantages/disadvantages. The reviewed concentration techniques are categorized into three major groups according to the concentration mechanisms involved.

The first group of concentration techniques termed as stacking methods is based on the difference in analyte velocity within two different moving buffer zones when analytes migrate from a faster velocity zone into a slower velocity zone and so being concentrated. Among the sample stacking techniques, one widely used is the sweeping method, in which analytes are incorporated into the micelles when micelles in the running buffer penetrate the sample zone and their mobility is considerably decreased in the stacking zone. However, the sweeping method is useful only for small hydrophobic analytes with a high affinity to a mobile micellar phase. For the field amplified sample stacking (FASS), the change of velocity is produced by low- and high-conductivity buffers. The concentration could be fairly high with FASS, but multi buffers are required. Isotachophoresis (ITP) is a technique using variable ion mobility zones for sample focusing and separation. Both ITP and FASS commonly need relatively long channels to preconcentrate larger sample volumes. Moreover, in sample stacking processes, the concentration enhancement is limited by the magnitude of analyte velocity change ratio, and also it is not easy to control the concentrated samples due to their motion.

The second group of analyte concentration methods is termed as field gradient focusing methods, in which analytes are focused at a unique equilibrium point where analyte net velocity is zero. The concentration enhancement in focusing techniques is determined by the rate at which analytes are transported to the focusing zone and the duration of the application of the focusing field, allowing for higher concentration enhancement in comparison with the stacking methods. Isoelectric focusing (IEF) technique is frequently used for the concentration and separation of proteins. In IEF, analytes is focused at their respective isoelectric points along a pH gradient. IEF however has limited applications since it is only applicable to analytes that have an accessible isoelectric point. In addition, most proteins are weakly soluble at their isoelectric points, which severely limits the extent of protein focusing with IEF. Electric field gradient focusing (EFGF) technique utilizes electric field gradient to suppress the net analyte velocity at a point in a channel where analyte focusing is achieved. In EFGF, the electrophoretic velocity gradient is created by manipulating electric field in the channel through an arrangement of electrodes, which however involves complex design and fabrication processes. In the family of field gradient focusing methods, temperature gradient focusing (TGF) is relatively new. In TGF, the concentration of sample analytes is achieved by balancing the electrophoretic motion of analytes against the bulk flow of buffer solution. The electrophoretic velocity gradient is created by using a temperature gradient along the channel with an appropriate buffer. TGF allows the concentration and separation of charged analytes in simple microfluidic structures with relatively short microchannels. Furthermore, TGF does not involve multi-buffers, embedded electrodes, membranes or nanostructures. Specifically, Joule heating induced TGF which has compact structure design can achieve the possibly high concentration enhancement in a short time, making it well suited for the development of integrated microfluidic systems.

The third group of analyte concentration methods utilizes various trapping mechanisms, such as electrokinetic trapping, dielectrophoretic trapping, immunocapture based trapping, magnetic beads assisted trapping and thermophoretic trapping. Electrokinetic trapping relies on the force due to ion concentration polarization for trapping analyte particles, dielectrophoretic trapping and thermophoretic trapping use dielectrophoretic force and thermophoretic force, respectively. Immunocapture based trapping utilizes the immunochemistry to immobilize analytes of interest on solid substrates. Magnetic beads assisted trapping uses magnetic force to trap the samples which are magnetized by adsorption of magnetic nanobeads. Usually, electrokinetic trapping performs much better than dielectrophoretic and thermophoretic trapping. Immunocapture based trapping and magnetic beads assisted trapping are specially used to concentrate biological cells, mostly CTCs. Some other emerging microfluidic concentration techniques are also briefly discussed.

**Figure 15:**
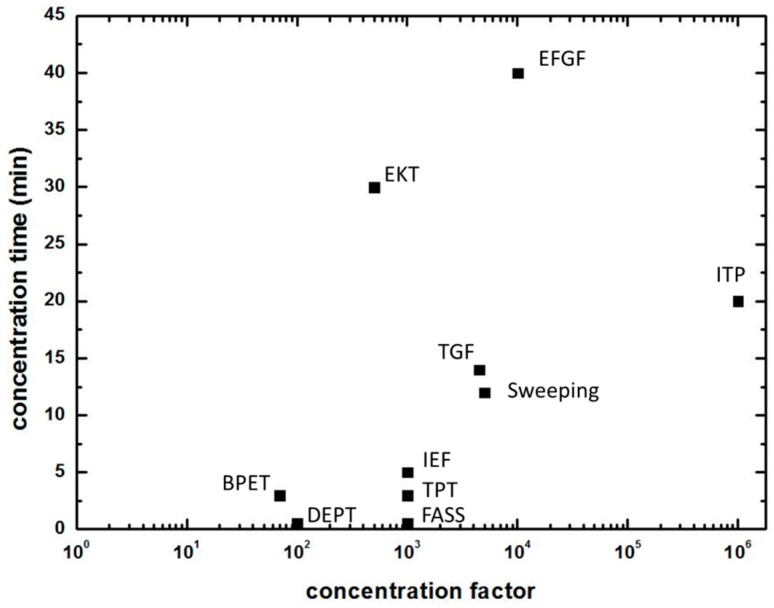
Concentration enhancement factor vs. concentration time for a variety of microfluidic sample concentration techniques. TGF, temperature gradient focusing (with Joule heating); IEF, Isoelectric focusing; EFGF, electric field gradient focusing; FASS, field amplified sample stacking; ITP, isotachophoresis; EKT, electrokinetic trapping; DEPT, dielectrophoretic trapping; TPT, thermophoretic trapping; BPET, bipolar electrode trapping.

Finally, here we provide a more quantitative comparison for the reviewed techniques. Figure 15 presents the concentration factor vs. concentration time for various microfluidic concentration techniques reviewed in the present work. The concentration factors in this figure correspond to the highest values documented in the literature. The concentration methods located on the bottom right of the figure suggests a better concentration performance, indicating a higher concentration factor achieved in a shorter time. This plot in combination with characteristics of each technique (such as application background, advantages and limitations) discussed above can be used to guide the choice of microfluidic concentration techniques for specific applications.
